# Surface-enhanced Raman spectroscopy: benefits, trade-offs and future developments

**DOI:** 10.1039/d0sc00809e

**Published:** 2020-04-14

**Authors:** Ana Isabel Pérez-Jiménez, Danya Lyu, Zhixuan Lu, Guokun Liu, Bin Ren

**Affiliations:** State Key Laboratory of Physical Chemistry of Solid Surfaces, Collaborative Innovation Center of Chemistry for Energy Materials (iChEM), College of Chemistry and Chemical Engineering, Xiamen University Xiamen 361005 P. R. China bren@xmu.edu.cn; State Key Laboratory of Marine Environmental Science, Fujian Provincial Key Laboratory for Coastal Ecology and Environmental Studies, Center for Marine Environmental Chemistry & Toxicology, College of the Environment and Ecology, Xiamen University Xiamen 361102 China guokunliu@xmu.edu.cn

## Abstract

Surface-enhanced Raman spectroscopy (SERS) is a vibrational spectroscopy technique with sensitivity down to the single molecule level that provides fine molecular fingerprints, allowing for direct identification of target analytes. Extensive theoretical and experimental research, together with continuous development of nanotechnology, has significantly broadened the scope of SERS and made it a hot research field in chemistry, physics, materials, biomedicine, and so on. However, SERS has not been developed into a routine analytical technique, and continuous efforts have been made to address the problems preventing its real-world application. The present minireview focuses on analyzing current and potential strategies to tackle problems and realize the SERS performance necessary for translation to practical applications.

## Abstract 2

Surface-enhanced Raman spectroscopy (SERS) is a vibrational spectroscopy technique with sensitivity down to the single molecule level that provides fine molecular fingerprints, allowing for direct identification of target analytes.

## Graphical abstract

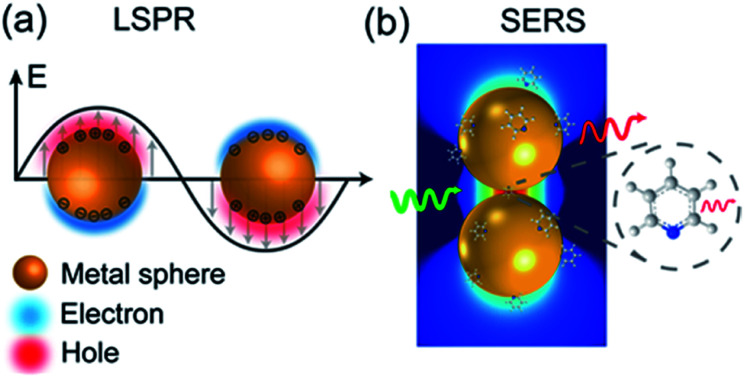

## 1. Introduction

Surface-enhanced Raman scattering (SERS) exploits the capability of metallic nanostructures to concentrate electromagnetic energy *via* optical modes called surface plasmons (SPs).^1^ The first observation of SERS occurred with the unexpected increase of the Raman signal from adsorbed pyridine on an electrochemically roughened silver electrode.^2^ The signal increase was originally attributed to a higher number of adsorbed molecules with the increased surface area, but it was later found to arise from anomalous surface enhancement.^3,4^ This discovery is of central importance for analytical purposes since it provides a means of overcoming the intrinsic low efficiency of the ordinary Raman scattering processes (d*σ*_R_/d*Ω* ∼ 10^−31^ cm^2^ sr^−1^) compared with those of fluorescence emission (d*σ*_F_/d*Ω* ∼ 10^−16^ cm^2^ sr^−1^) and infrared absorption (d*σ*_IR_/d*Ω* ∼ 10^−20^ cm^2^ sr^−1^). Therefore, the technique developed on the basis of the phenomenon, *i.e.*, surface-enhanced Raman spectroscopy (also abbreviated as SERS) enables the examination of small numbers of (or even single) molecules. In addition to its high sensitivity, SERS inherits from Raman spectroscopy characteristics like chemical specific (fingerprint), non-destructive, and label-free analytical nature. Thanks to these attributes, SERS has caught the attention of different research communities, and the past four decades have witnessed a dramatic increase in its applicability.

Nevertheless, SERS may appear challenging for many newcomers as there is an apparent gap between the high-quality performance shown in numerous publications and the relatively low practical performance in the detection of real samples. Therefore, it is urgent for the SERS community to address the problems preventing the progress of SERS from a promising technique to a practical one. Further effort is encouraged to improve the data reproducibility, substrate stability, and substrate–analyte interactions.^5–9^ From our point of view, these issues could be solved by proper control of the experimental conditions.

Therefore, we offer a systematic yet concise analysis of the strategies for addressing these challenges, and we demonstrate how these strategies can push SERS to become a viable technique for quantitative analysis. In addition, we share our vision of some directions that could improve the performance of SERS for practical applications. We present this minireview in three parts. First, we provide a brief description of SERS mechanisms and surface selection rules. Then, we offer a detailed discussion of the origin of SERS shortcomings and potential solutions, and we provide a routine for quantitative measurement. Last, we address future developments of SERS, including pushing to the limit of sensitivity and energetic, spatial, and temporal resolution.

## 2. SERS mechanisms and surface selection rules

### 2.1 SERS mechanisms

With respect to the ordinary Raman spectroscopy, the main outcome of SERS is the gigantic enhanced Raman intensity in the level of several orders of magnitude, which is widely agreed mainly from the joint contribution of electromagnetic and chemical mechanisms. To better explain this, we start by describing the basic aspects of the Raman scattering effect. We then demonstrate how the excitation and scattering processes can be modified by the above enhancement mechanisms, which lead to the SERS effect.

The classical theory of Raman scattering establishes that a molecule under an oscillating incident electric field may experience an induced polarization (*P*_0_) and emit scattered light at a Raman-shifted frequency (*ω*_R_).^10,11^ The magnitude of *P*_0_ depends on the Raman polarizability of the electrons in the molecule (*α*^R^_0_) and the strength of the incoming electromagnetic radiation *E*_0_ (with a frequency of *ω*_0_), as follows:

1 *P*_0_(*ω*_R_) = *α*^R^_0_(*ω*_0_,*ω*_R_)*E*_0_(*ω*_0_)

Qualitatively, *α*^R^_0_ represents the change in the electron cloud during the molecular vibration, and the Raman scattering involves the interaction between two basic elements: a molecule and an incoming radiation. On the other hand, for SERS to occur, it requires the presence of metallic nanostructures. Therefore, a complete description of SERS involves the interaction among light, molecule, and metallic nanostructure.^12^

**Fig. 1:**
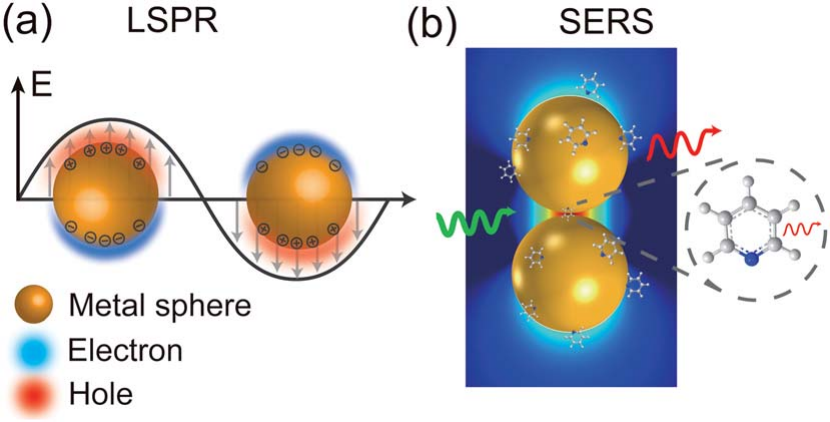
Schematic diagrams of (a) LSPR effect: metal conductive electrons are excited into collective oscillations generating an electromagnetic field highly localized in the metal–dielectric interface when irradiated with light; (b) nanoparticle–molecule interaction: leading to the mutual excitation of the Raman polarizability (red thin arrow) from the local EM field (green arrow) and generating the enhanced Raman signal of molecule (thick red arrow). Reproduced with permission from ref. 14 Copyright 2016 Nature Publishing Group.

When a metal nanostructure is irradiated with an incoming light (*E*_0_), the conductive electrons are delocalized into collective oscillations, which generate an EM field around the interface formed by the metal nanostructure and the dielectric environment (Fig. 1a). The frequency of such electron oscillations depends on the density of electrons, the effective electron mass, and the shape and size of the charge distribution.^13^ If the frequency of the incoming radiation (*ω*_0_) is resonant with that of the electron oscillation, the excitation process is referred as surface plasmon resonance (SPR). SPR can either propagate as a longitudinal wave at extended metal surfaces or remain highly localized on places such as edges, tips, or crevices of the interface between the metallic surface and the dielectric, corresponding to surface plasmon polaritons (SPPs) and localized surface plasmon resonance (LSPR), respectively.^14^ Generally, the local EM field (*E*_loc_) associated with the LSPR is higher in magnitude than the incident EM field, which results in the enhancement of the EM field in SERS by a factor of *G*_ex_ = [*E*_loc_(*ω*_0_)/*E*_0_(*ω*_0_)]^2^.

Similarly, other sources of oscillation, such as dipoles (*i.e.* modified Raman dipole, *P*_0_) or quadrupoles lead to the excitation of the LSPR. Typically, the Raman polarizability of molecules interacting with metal nanostructures (*P*_0_) are about one or three orders of magnitude larger than that of free molecules. Therefore, the interaction of molecules with the vicinal metal nanostructures leads to the mutual excitation of the Raman polarizability by the local EM field, and *vice versa* (Fig. 1b). In this process, the EM field in SERS is enhanced by a factor of *G*_R_ = [*E*_loc_(*ω*_R_)/*E*_0_(*ω*_R_)]^2^. For Raman modes with low vibrational frequency, *ω*_0_ can be considered roughly equal to *ω*_R_, and the EM field enhancement factors *G*_ex_(*ω*_0_) and *G*_R_(*ω*_R_) are comparable. Therefore, the overall enhancement of the EM field in SERS (*G*) scales with the fourth power of the local EM field enhancement,^14^ as follows:2
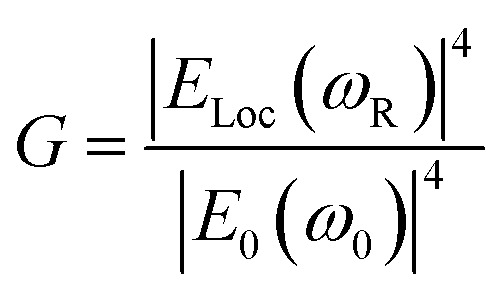


Therefore, a minor change in the local field enhancement can lead to significant variation in SERS enhancement. Moreover, when two nanoparticles are spaced 1 nm apart, the plasmons of the two nanoparticles can couple with each other. In this case, the SERS signal of one molecule can be enhanced by as high as 9 to 12 orders of magnitude when it is placed in the 1 nm^3^ volume of the gap between the two nanoparticles (the so called hot spot), and it may decay quickly with increasing distance from the hot spot. Therefore, the molecules adsorbed inside and outside the hot spot will give very different Raman intensities. Up to now, it is still impossible to precisely control the distance between nanoparticles. These two effects lead to the low reproducibility in SERS measurements.^15–17^

Conversely, the chemical mechanism encompasses effects that result from molecular adsorptions and lead to the modification of *α*^R^_0_^18^ as a result of either chemical complexation, or charge transfer and charge transfer resonance.^18,19^ Chemical complexation refers to the formation of an adsorbate-metal nanostructure complex which may lead to a change in the Raman polarizability, spatial orientation, and symmetry of the adsorbed molecule in comparison with its free state, promoting the possibility of certain vibrational modes (*vide infra*). The presence of charge transfer or charge-transfer resonance implies electronic coupling between the metal and the molecule. In the case of the charge transfer process, there is an exchange of electrons between the Fermi level of the metal and the lowest unoccupied molecular orbital (LUMO) or highest occupied molecular orbital (HOMO) of the molecule. Charge-transfer resonance occurs when the laser wavelength matches the electronic transitions of the adsorbate-metal complex, resulting in the enhancement of the Raman scattering through the resonance Raman effect (RRS).^20^ It is worth mentioning that the chemical mechanism may result in either a quenching or an enhancement of the scattering process.^11^ In addition, its expected contribution to the overall SERS enhancement is usually not higher than a factor of 10^3^, which is much lower than the 10^5^ to 10^9^-fold contribution obtained from the EM mechanism.^14^

On considering the two types of enhancement mechanisms, the Raman dipole under SERS conditions (*P*) can be defined as:

3 *P*(*ω*_R_) = *α*^R^(*ω*_0_,*ω*_R_)*E*_loc_(*ω*_0_)

where *α*^R^ is the modified Raman polarizability, and *E*_loc_ is the enhanced local EM field around the surface of the metallic nanostructure as explained above.

### 2.2 Surface selection rules

The Raman spectral profile from a “free” molecule can be simply predicted by applying symmetry and point group theory. For SERS, the prediction and interpretation of the spectral profile are usually not that straightforward. First, the local EM field in the visible region has components perpendicular and parallel to the nanostructure surface as a result of the interaction of light with a plasmonic nanostructure. Second, the molecule may take a certain orientation at the nanostructure surface when it interacts with the nanostructure. For a vibrational mode to achieve a high SERS enhancement, it requires the mode to have a polarizability change along the direction of the local EM field. This is the basis of the concept known as surface selection rules, which describe the corresponding symmetry properties of the modified Raman dipole (*P*) and changes in the relative intensities of the Raman peaks caused by the EM field polarization at the metal surface.^21,22^ Furthermore, surface selection rules allow the understanding of the selective enhancement of certain vibrational modes because of the preferred orientation of the adsorbed molecule on the nanostructure surface.

## 3. Key factors to boost SERS

### 3.1 Reliable estimation of SERS enhancement factors

SERS enhancement factor (EF) is an essential parameter for evaluating the analytical performance of a SERS substrate, and it is defined as the ratio of SERS intensity contributed by each surface molecule to the ordinary Raman intensity contributed by each free molecule. EF is normally estimated by the average SERS enhancement:^23^4
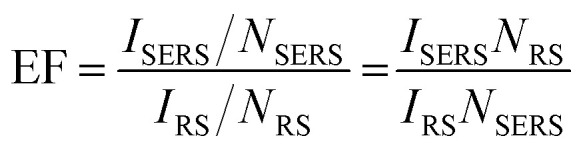
where *I*_SERS_ and *I*_RS_ are the SERS and ordinary Raman intensities, respectively, which can be obtained experimentally. It is better to use the peak area to obtain *I*_SERS_ and *I*_RS_, especially when the peak widths of SERS and ordinary Raman are different. It is quite common that the full width at half maximum (FWHM) of a SERS peak is broader than that of the ordinary Raman peak due to the more heterogeneous state of molecules on surface, except those molecules having strong and complicated interaction (such as hydrogen bonding) with the surrounding molecules. *N*_SERS_ and *N*_RS_ are the numbers of molecules probed by SERS and ordinary Raman,^19,23–26^ and they require careful consideration to obtain the correct values. Experimentally, there are two ways to obtain *I*_RS_, *i.e.*, on solid sample dried from solution, or in solution, where different methods are applied to estimate *N*_RS_.^7^

**Fig. 2:**
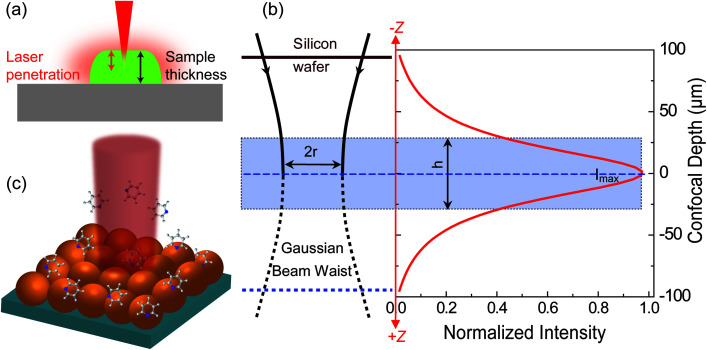
Schematic diagrams of (a) the limited penetration depth of a laser beam through a dried sample (especially to one with a high concentration of fluorescent dyes), due to the strong absorption at the laser wavelength; (b) the waist profile of the laser beam in solution, the corresponding confocal depth (*z*) and the Raman intensity–depth profile calculated from the integrated intensity of the 520.7 cm^−1^ for a single crystal Si wafer; reprinted with permission from ref. 7. Copyright 2009 Springer; (c) the excitation configuration during SERS measurement, where the number of target analytes contributing to SERS is determined by the surface coverage of adsorbed targets rather than the concentration in solution, and the average SERS signal from multiple spots may report a more consistent EF value due to the non-uniform distribution of molecules over the SERS substrate.

For estimating *I*_RS_ in dried samples, a drop of solution with known concentration (*C*_RS_) is placed on a substrate and let dry. With the known density (*ρ*) and thickness (*t*) of the dried sample and the laser spot radius (*r*), *N*_RS_ contributing to the ordinary Raman signal can be estimated using *N*_RS_ = *ρ*π*r*^2^*t*. It should be noted that *t* is quite frequently overestimated in the literature, and hence the EF is also overestimated. The main reason is that the most commonly used Raman probes are dye molecules, which have strong absorption at the laser wavelength. Therefore, the penetration depth of the laser in the sample is usually smaller than *t*, the thickness of the solid dye sample (Fig. 2a).

For estimating *I*_RS_ using liquid samples, a solution of known concentration (*C*_RS_) is measured and the number of molecules is determined by the relation *N*_RS_ = *C*_RS_*V*.^7^ Due to the remarkable depth-sensitive resolution of confocal microscopes, the collection efficiency varies with the confocal depth, and thus not all the molecules within the probing volume contribute equally to the overall Raman signals. This results in an overestimation of the number of molecules effectively illuminated in the scattering volume.^25^ We proposed a method to obtain the effective volume (*V* = *Ah*, where *A* is the area of illumination or laser-spot size) from which all the molecules contribute equally to the overall Raman signal (Fig. 2b). *h* represents the thickness of an ultrathin film or solution layer near of the “ideal focal plane” (*z* = 0, where the Raman intensity is the highest), and it can be obtained by the following steps. The first step is to obtain the confocal depth profile by immersing a standard sample (single-crystal Si wafer is recommended) in a solution and then collect the Raman signal (the peak for Si is at 520.7 cm^−1^) arising from different planes located at certain distances (*z*) below and above the focal plane (*z* = 0). Like that, the overall signal can be obtained by integrating the signal over the confocal depth profile 
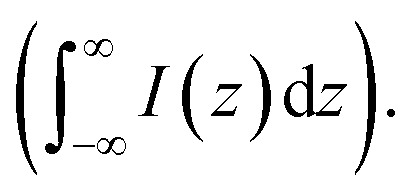
 Then, *h* can be calculated by:5
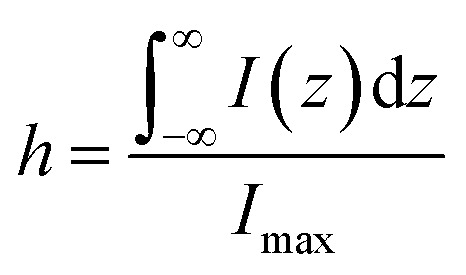


Practically, the *h* value is determined by the pinhole size and the numerical aperture (NA) of the objective. Importantly, the optical configurations for collecting SERS and ordinary Raman signals must be identical. Otherwise, the results will be prone to error.

*N*_SERS_ can be calculated by relating the known geometric surface area (*A*) in the illuminated spot with the surface area (*σ*) occupied by the molecule assuming full monolayer adsorption, as follows:6
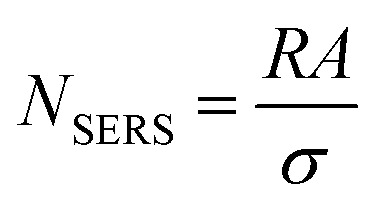


Some important aspects must be considered when calculating *N*_SERS_: (1) selecting a suitable target analyte (*i.e.*, a molecule with a well-defined Raman scattering cross-sections) to facilitate the analysis;^27^ (2) ensuring surface cleanliness to avoid possible competing species; (3) the surface coverage (*θ*) must be restricted to a single monolayer to estimate precisely the number of molecules on the surface to avoid a misleading concentration effect on EF calculation. It is a common practice to determine *N*_SERS_ by adding droplets of known concentration and volume to the solid SERS substrate (Fig. 2c) or mixing with the colloid in solution directly. In both cases, the surface coverage (*θ*), rather than the concentration, must be used for the reliable calculation of *N*_SERS_. Meanwhile, it is highly recommended to analyse multiple spots of the substrate when measuring *N*_SERS_, and the average value is preferred to report a more consistent EF value.

If correctly estimated, the EF could be used as the critical parameter to evaluate the SERS sensitivity from different SERS substrates. One should bear in mind the following four points. (i) The evaluation should be under the same conditions, especially with the same analyte under the same surface coverage. (ii) The analyte has no resonance Raman effect under the applied laser wavelength. (iii) The EF may vary when different analytes are used, due to the different strength of interaction between the analyte and the SERS substrate. Therefore, selecting the right probe molecule is the first step for calculating the average EF.^27^ (4) The lowest detectable concentration of the analyte is more meaningful than the EF for practical applications.

### 3.2 Reproducibility

Specifically, reproducibility of SERS signals refers to the fact that under similar test conditions, read-out signals from multiple tests may be comparable within a certain error range (20% is acceptable if commercial substrates are used^5^). However, SERS has been criticized for its poor reproducibility in practical applications, mainly due to the high heterogeneity of the EM enhancement. In addition, in practical experiments, many other factors, including instrumental conditions and sample preparation, may also affect the reproducibility of SERS signals.^28^ Therefore, it is important to analyse the origins of these factors and determine ways to mitigate their impacts.

#### 3.2.1 Instrumental conditions

Drift in the optical alignment and subtle changes in either sample position or laser power density can cause spectral intensity variations not related to the concentration of the analyte.^28,29^ Therefore, it is very important to routinely calibrate the Raman instrument, so that its spectral position and intensity are under standardized conditions.^30^ One may consider to increase the size of the laser spot to larger than 10 μm by using either a low NA objective^31,32^ or under-filled or slightly non-collimated laser beam. In this way, more reproducible SERS signals can be obtained due to averaging of a larger number of molecules in the excitation volume.

#### 3.2.2 Selection of materials for SERS substrates

Any material supporting plasmon activity at the excitation wavelength can serve as a SERS substrate, as long as it shows good chemical stability in the applied environment. For example, the oxidation of the surface may significantly change the LSPR properties of the SERS substrate and its interaction with the analyte, which will significantly influence the reproducibility of SERS substrates.

Gold, silver and copper are the most commonly used materials for SERS substrates with outstanding SERS enhancements. Silver can be used for excitation over the whole visible to near infrared region, whereas gold and copper are used for the red and near infrared regions. Silver has a higher plasmon quality than that of gold and copper, and gold has a much better chemical stability.^33^ In some specific applications such as catalysis and electrochemistry, transition metals like platinum, palladium, iron, nickel and their alloys with a low SERS enhancement have to be used.^34,35^ Among them, platinum and palladium are chemically robust, whereas iron and nickel are prone to corrosion. Alkali metals are highly reactive in air and aqueous environments, so they can be only used in vacuum^36^ or potentially in non-aqueous media. Aluminium is commonly used for applications in the ultraviolet region,^37,38^ but easily oxidized in air. There are increasing application of using semiconducting and dielectric materials (metal oxide, MOs) as SERS substrates,^39–41^ which display high stability, but the SERS enhancement is weak and enhancement mechanism is still unclear.^42–46^

#### 3.2.3 Uniformity of substrate

A critical step for ensuring the reproducibility of SERS signals is the fabrication of SERS substrates with well-controlled nanostructured features. With the development of nanotechnology, diverse methods have been proposed for preparing SERS substrates.^7^

The electrochemical oxidation and reduction cycle(s) (EC-ORC)^2^ method was often used to prepare SERS substrates in the early stage of SERS. It does not require the use of reducing or protecting agents and thus the obtained substrates have very good stability and clean surfaces.^34,47^ However, their surfaces usually have random nanostructured features with a wide size and shape distribution.

Over the past 20 years, remarkable efforts have been made to develop nanostructures, such as nanospheres, nanorods, nanocones, cubes, triangular plates, and octahedra, with well-defined morphologies and size distributions by sol–gel synthesis.^48^ Frequently, protecting agents, like citrate ions, CTAB, and PVP, are employed to control the crystal growth and dispersity of the nanoparticles in colloidal solution. These structures form the basic building blocks for further SERS applications in solution or for the fabrication of a solid substrate.

Solid substrates are more convenient to use compared with colloidal solutions. They can be prepared by self-assembly of the abovementioned nanoparticles on solid supports. Possible interference from SERS signals of the residual protecting agent and reducing agent from the synthesis step should be eliminated, for example, by modifying the surface of the gold or silver nanoparticles with iodine ions^49^ or spermine.^50^ Substrates prepared by template and photolithography methods are usually clean and high ordered, showing very good uniformity.^51–53^ Most recently, SERS substrates with an area of the wafer scale and high enhancement have been prepared by using photolithography methods.^54^

#### 3.2.4 Stability of solid substrates

Another central aspect concerning the SERS reproducibility is the inherent structural instability of the hot spots on SERS substrates. The hot spots may undergo structural changes *via* melting^55–57^ or diffusion of surface atoms^58,59^ induced or accelerated by the laser illumination, resulting in a change of the shape, size, and interparticle distance of the nanoparticles.^60^ Diffusion of surface atoms is a consequence of their lower coordination number compared with interior atoms.^61,62^ Nanoparticles with anisotropic morphologies like nanorods, nanostars,^63^ and nanocubes, which show high EFs, are more prone to surface diffusion to decrease their surface energy by reshaping the nanoparticles into a more stable structure (spherical shape). This effect many be avoided by decreasing the laser power density and/or exposure time^64^ or modifying the surface with the shells of other metals, metal oxides, or strong adsorbates.^65–67^

#### 3.2.5 Stability of colloidal substrates

The SERS measurement in colloidal solution usually shows a better reproducibility than that on the two-dimensional solid substrate due to the average effect of millions of hot spots in the three-dimensional detection volume compared with the small detection area in the two-dimensional solid substrate. Nevertheless, factors like surface chemistry, charge, adsorbed molecule and external environment, may compromise the stability of the nanoparticle colloids.^68^ Especially, when aiming at the ultrahigh sensitive detection towards ultralow concentrated target, the detected SERS signal may fluctuate constantly due to the dynamic interaction between target and colloidal nanoparticles, but still better than the condition in solid substrate. Therefore, it is important to understand the factors governing the behaviour of the colloidal nanoparticle to ensure the effective formation and control of nanoparticle aggregates.^69^ Techniques like dynamic light scattering (DLS),^70^*in situ* transmission electron microscopy (TEM),^71^ high-sensitivity flow cytometry (HSFCM)^72^ and zeta potential measurements^73^ are useful for measuring the state of colloids during the dynamic aggregating process. Meanwhile, the theoretical approach known as the extended Derjaguin, Landau, Verwey, and Overbeek (x-DLVO) model can be used to determine and predict the state of nanoparticle colloids and buffer compatibility.^74^ It could be valuable if the SERS community adopts this theoretical method to have a prediction for consistent SERS signals.

Microfluidic devices represent an alternative strategy for improving the SERS reproducibility of colloidal nanoparticles by: (1) enabling the precise control of the aggregation time using the strategy of replacing time with space; (2) reducing the mechanical and optical variations of the colloids; (3) making more efficient mixing with the analyte (automation); (4) decreasing the likely contamination (isolated systems).^75–77^

#### 3.2.6 Shelf life

Another important aspect in the stability of SERS substrates lies in the shelf life, if their commercialization is desired. For colloidal substrates, increasing the storage time will increase the probability of nanoparticle flocculation due to physical collisions.^61^ It is necessary to control the surface modification (by capping agent or protective layer *via* electrostatic repulsion or by stereo protection), the solution media, and the concentration to eliminate the flocculation process and thus to improve the stability of nanoparticles. In comparison, solid substrates are structurally more stable during storage than colloidal substrates. However, contamination or deterioration may occur, and these should be prevented by vacuum protection.

### 3.3 Substrate–molecule interaction

A SERS spectrum is a sum of analyte signals and background noises originating from phenomena such as substrate photoluminescence^78^ and fluorescent or Raman signals from the solvent or impurities.^79,80^ Noise may dominate or overwhelm the SERS signals from the analyte when the analyte signal is weak, or the analyte concentration is extremely low. This problem may be alleviated by choosing an excitation wavelength in the near infrared region, which requires LSPR in the corresponding region.^81^

An alternative and better solution to the above problem is to directly improve the signal. From the viewpoint of the EM enhancement, the SERS signal is mainly contributed by the analyte located in hot spots, where the fluorescence signal is significantly quenched. Therefore, the number of hots spots, analyte molecules, and their locations within these plasmonic structures directly determine the strength of the SERS signal. Taking a dimer of gold nanoparticles as an example, the SERS signal is several orders of magnitude higher when a molecule is placed at the center of the hot spot compared with that outside the hot spot. Therefore, it will be ideal if the hot spot can be designed with ligands that can specifically capture the analyte molecules.^16^

Molecules bearing functional groups such as –SH, –NH_2_, –NH_4_^+^, –COO^−^, –CN, and carbonyl can strongly bind to metallic cores and replace capping agents.^82^ Thereby, the signal-to-noise ratio from the analyte can be improved with a longer detection time.^83^ However, a wide range of molecules have weak interactions with the substrate and can just transiently contact the substrate. Therefore, the diffusion of molecules into and out of the hot spot would lead to fluctuating (even on and off) SERS signals, especially at ultralow concentrations. A strategy for tackling this problem is to set the acquisition time as short as possible (usually in the milliseconds, limited by the frame rate of the detector). In this way, the spectra containing the largest contribution of the SERS signal of analyte can be selected and that containing only the background can be discarded.^80^ Thereby, we can minimize the interfering background and improve the signal to background ratio of SERS spectra.

**Fig. 3:**
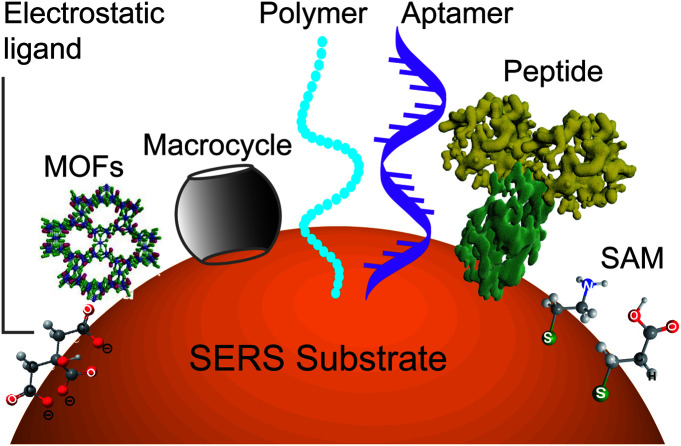
Scheme of diverse surface-affinity ligands, which immobilize analytes *via* either physisorption or chemisorption (covalent bond) interactions: electrostatic modification drives the adsorption of analytes on the surface of nanostructures by electrostatic force; molecular steric effects by porous materials like MOFs allow the selective permeation of small molecules *via* the molecular sieving effect; host–guest recognition by macrocycles or MIP captures a wide variety of guest molecules with a high affinity; biological recognition with biological polymers like DNA or peptides has a high affinity to diverse analytes with sizes ranging from small molecules to macromolecules or even cells; and chemical derivatization by a small bridge molecule on the surface with the functional group reacts with the analyte.

However, it will be better to capture the analyte on the surface throughout the detection time scale. This can be realized by improving the binding affinity between the analyte and the surface. A set of methods for modifying nanostructured surfaces with a layer of materials that immobilize analytes *via* either physisorption (*i.e.*, electrostatic, π interactions, hydrogen bond and van der Waals forces) or chemisorption (covalent bond) interactions have been used to improve the binding affinity and the molecular selectivity as well. We discuss some of these surface affinity strategies widely used in analysis (Fig. 3).

#### 3.3.1 Electrostatic modification

Electrostatic force can drive the adsorption of analytes on the surface of nanostructures. For example, citrate-capped gold nanoparticles present net negative charges, so these nanoparticles can attract positively charged dyes such as R6G, malachite green, and crystal violate *via* electrostatic forces and realize trace detection of these analytes.

#### 3.3.2 Molecular steric effect

Porous materials like metal organic frameworks (MOFs), covalent organic frameworks (COFs), and zeolites present channels and cavities. Their sizes fall within the range of that of the analyte molecules and allow the selective permeation of small molecules *via* the molecular sieving effect.^84–88^ These materials can be used as a coating layer over the SERS substrates for trapping the analytes within their pores or channels and driving them to the hot spots. This strategy is suitable for the analysis of samples, like small molecules and gas samples (*i.e.*, aldehydes), that can easily diffuse away from the metal surface.^85^

#### 3.3.3 Host–guest recognition

Macrocycles such as cyclodextrin, cucurbit[*n*]uril and calix[*n*]arenes have cylindrical-like cavity structures and can serve as high-affinity agents to capture a wide variety of guest molecules due to synergistic interactions.^89^ For example, cucurbituril can directly adsorb onto gold surfaces through multi-carbonyls-Au interactions and effectively encapsulate either positive or neutral compounds such as methyl viologen and ferrocene. It has been used to link two nanoparticles together to create a detection region at the center of the hot spot.^90,91^ Calixarene and cyclodextrin derivatized with thiol groups can be used for the detection of polycyclic aromatic hydrocarbons and antibiotics with a low affinity to metals.^92,93^ Macrocycles with a small size (less than 3 nm) and synergistic affinity can achieve a small target-surface distance to enhance the signal of the analyte. It should be noted that usually a small host can only effectively accommodate low-weight molecules or ions and has difficulty capturing large molecules, like antibodies or long polymers. It will be helpful to use the synergistic effects of multiple small or orthogonal ligands to recognize large analytes.^94,95^ Molecular imprinted polymers (MIP) can capture molecules into their cavities in a similar way to that used for host–guest interaction. However, it is extremely difficult to completely remove the template from the imprinted polymer network during the imprinting step, which may lead to false positives due to the presence of residues. Furthermore, some of them have complicated SERS spectra which may cover the signals of analytes.^96^

#### 3.3.4 Biological recognition

Biological polymers like DNA or peptides present specific structures that allow them to have a high affinity to diverse analytes with sizes ranging from small molecules to macromolecules or even cells. Thanks to their structure-related recognition, the recognition process can be achieved with binding affinities down to the nM or even pM level.^97,98^ The related experiments should be carefully handled, since the functional structures of DNA and peptides depend very much on the surrounding environmental factors such as temperature, pH and ionic strength.^99,100^

#### 3.3.5 Chemical derivatization

Chemical derivatization is another way to capture the analyte onto the surface and to be detected. Usually, a small bridge molecule is covalently bound to the metal surface with the functional group facing outward to react with the analyte. For instance, gold nanoparticles modified with cysteine have been used for the detection of 2,4,6-trinitrotoluene (TNT) *via* the formation of a Meisenheimer complex between cysteine and TNT, which further induces the aggregation of nanoparticles to form hot spots.^101^ If this kind of chemical derivatization can produce surface species with resonance Raman effect, it can significantly enhance the signal. For example, the derivatization of bisphenol A with aryl-diazonium leads to a limit of detection as low as 10 amol.^102^

### 3.4 Optimizing SERS for quantitative analysis

The fingerprinting and ultrasensitive merits of SERS have positioned this technique as a powerful qualitative analytical tool. Nevertheless, despite of several works endorsing its quantitative capabilities, this characteristic is not well-recognized as a signature of SERS. Intrinsically, as a linear spectroscopic method, Raman spectroscopy possesses the same level of quantitative analytical ability as other well-accepted spectroscopic techniques, including UV-Vis/IR absorption and fluorescence. However, the use of SERS for quantitative analysis is challenging due to the complicated interactions between light, molecule, nanostructure, and plasmon. Huge efforts have been made to develop SERS into a quantitative tool in the SERS community since the discovery of SERS four decades ago.^8,12,27,103,104^

**Fig. 4:**
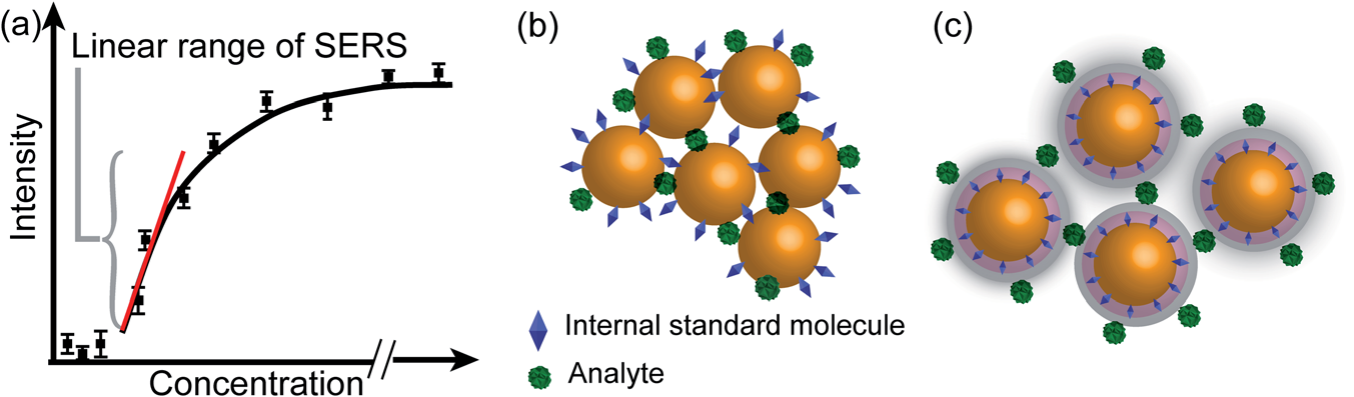
Scheme of three strategies for pushing the limit of quantitative detection. (a) Direct extraction of the semi-quantification linear range (red line) from the plot of SERS intensity *versus* analyte concentration (black curve). (b) Improved quantification by introducing an internal standard with the simultaneous adsorption mode. (c) Core-molecule-shell mode with the internal standard embedded between the core and shell of nanoparticle to avoid the potential competitive adsorption between the internal standard and analyte.

In SERS, only the molecules staying on the enhanced surface can experience the strongest enhancement. Therefore, it is inherently a surface-specific technique. The relation between SERS intensity and analyte concentration (specifically, surface coverage) has to be analyzed with adsorption models including Langmuir, Freundlich, Tempkin or Brunauer–Emmett–Teller isotherms, which may complicate the analysis.^83^ For instance, according to the Langmuir adsorption model (Fig. 4a), the analyte surface coverage follows the relation 
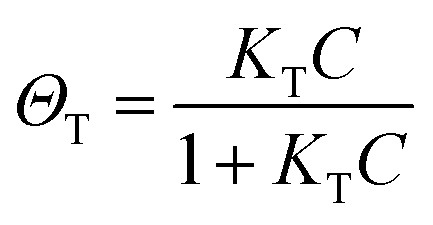
, where *Θ*_T_ represents the surface coverage, *K*_T_ represents the adsorption equilibrium constant, and C represents the concentration of molecules in solution.^105^ The SERS intensity (*I*_T_) of the adsorbed analyte can be represented as follows:

7 *I*_T_ = EF_T_*I*_T0_*Θ*_T_*S*_T_*N*_T_

where, EF_T_ corresponds to the average SERS enhancement factor of the analyte, *I*_T0_ corresponds to the ordinary Raman intensity of the analyte, *S*_T_ represents the active SERS surface area and *N*_T_ represents the analyte surface density at the highest molecular packing. It is a common practice in quantitative analysis to compare the signals collected from a sample with those of standard solutions of the same molecule (known as calibration curve).^106^ However, in practical analysis, the absolute *I*_T_ varies with the measuring condition and the aggregation state of the SERS substrates, which makes it difficult to obtain reliable quantification.

It is advisable to use an internal standard to overcome the above issues. Like eqn (7), the intensity of the internal standard (*I*_IS_) can be represented as:

8 *I*_IS_ = EF_IS_*I*_IS0_*Θ*_IS_*S*_IS_*N*_IS_

where, EF_IS_ represents the average SERS enhancement factor of internal standard, *I*_IS0_ represents the ordinary Raman intensity of the internal standard, *Θ*_IS_ is the surface coverage of the internal standard, *S*_IS_ represents the active SERS surface area of the internal standard, and *N*_IS_ represents the internal standard density at the highest molecular packing.

We can obtain the corrected intensity (*I*_R_) by normalizing the signal of analyte molecules *I*_T_ with that of the internal standard:9
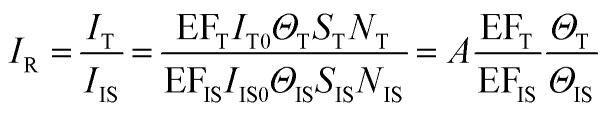
where the constant *A* is equal to *I*_TO_*S*_T_*N*_T_/*I*_IS0_*S*_IS_*N*_IS_. By a careful experimental design, the analyte and internal standard enhancement (EF_T_/EF_IS_) remains constant, and the surface coverage of the internal standard remains unchanged. Therefore, *I*_R_ will have a linear relationship with the surface coverage of the analyte (*Θ*_T_). Moreover, since any source of interference simultaneously affects the signal intensities of both the analyte and the standard, taking the ratio between the signal of the analyte and the internal standard may suppress the interference.^29^

The internal standards are required to be in the same physical and chemical environment with the analyte molecules (Fig. 4b), which is challenging for experimentalists, since SERS measurements are conducted with a multi-phase system, and SERS intensity depends on the highly localized near field enhancement. Various strategies have been applied for building the right internal standard for a specific analyte. For example, one approach is to use internal standards by coating the SERS substrate with a layer of molecules (such as alkane thiols or analyte-capture ligand) that can strongly interact with the SERS substrates, or adding the isotope edited versions of the analyte with known concentration into the solution.^96,107,108^ However, even if the structural analogue of the analyte molecule is used as the internal standard, so that the internal standard and analyte molecules can be almost equally adsorbed on the SERS substrate in an indiscriminate microenvironment, the following issues are still to be taken into account: (1) the dynamic exchange and competitive adsorption between these two surface species are indispensable, especially when their concentrations are significantly different; and (2) the SERS signal of the internal standard may be influenced by the microenvironment, leading to a change in intensity and frequency.

The core-molecule-shell (CMS) approach has been proposed as a solution to the above issues. In CMS, the molecules utilized as the internal standard are embedded between the nanoparticle core and the external shell^109^ so that the signal of the internal standard will not be influenced by the outer environment and the analyte molecules can occupy the full surface of the shell (*i.e.*, a constant *Θ*_IS_) (Fig. 4c). Because of this, the competitive adsorption and the dynamic replacement between the internal standard and the analyte is eliminated. In this case, eqn (9) can be expressed as:10
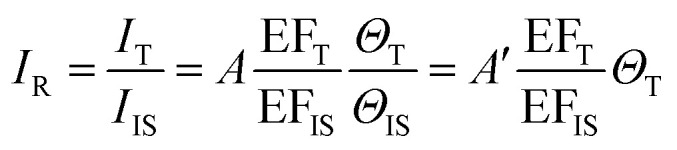


According to eqn (10), if the ratio between the analyte and internal standard enhancements (EF_T_/EF_IS_) is constant, *I*_R_ is linear with respect to the surface coverage of the analyte. This, in turn, expands the linear range of detection and meanwhile the internal standard molecules do not have to exhibit similar spectral and chemical properties to the analyte ones. Furthermore, the signal intensities of the analyte and internal standard can be made comparable to each other either by adjusting the surface coverage of the internal standard or by choosing an internal standard with a suitable Raman scattering cross section.

It should be noted that the CMS method is assumed to work when the enhanced fields on the surface and inside the gap of shell and core can be correlated. For the detection of extremely low concentrations, in which the analytes are not uniformly distributed over the shell surface, this requirement may not be fulfilled, and a specific calibration approach must be developed for this regime. One may also use a native internal standard in DNA detection. For example, the signal of phosphate groups from the DNA backbone can be applied as the internal standard to calibrate the signals from the nucleobases, providing that the relative content of nucleobases, rather than the concentration of the DNA, is important.^49^

### 3.5 Guidelines for the qualitative and quantitative SERS analysis

**Fig. 5:**
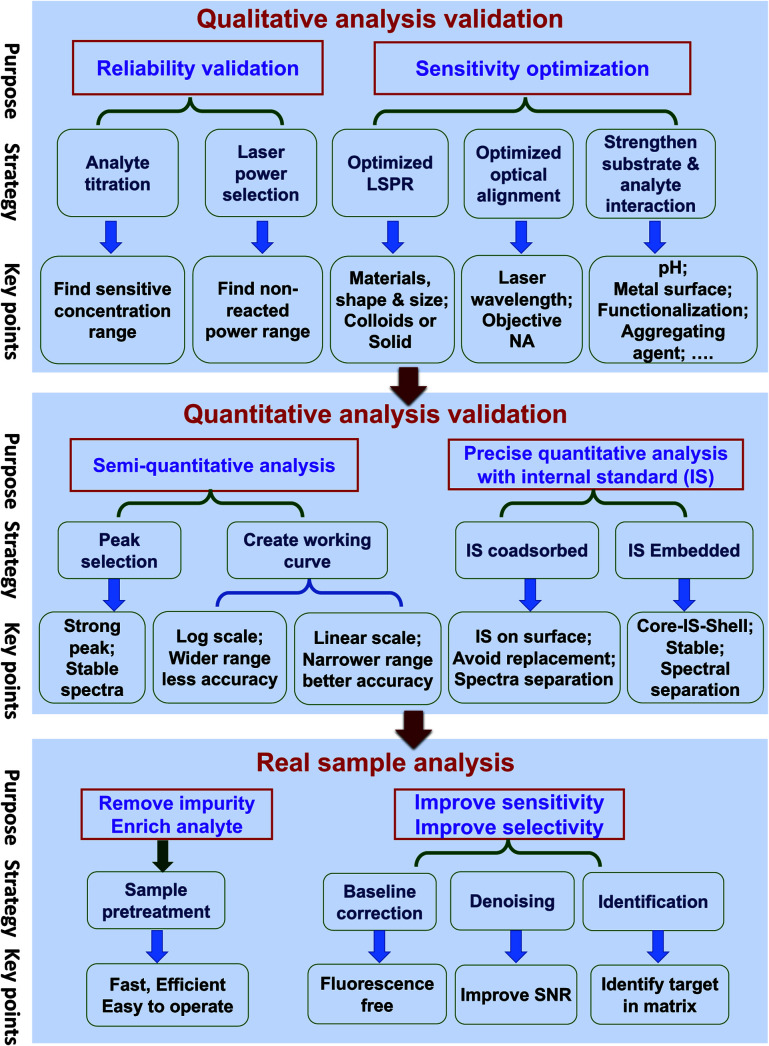
The three-step guideline towards the quantitative SERS analysis of real samples showing the purpose, strategy, and key points. The first step is to realize reliable qualitative analysis and optimize the detection sensitivity. The second step involves the creation of a working curve with the desired accuracy and the utilization of internal standards to further improve the accuracy. The third step is to deal with real samples enabled by sample pretreatments and data analyses.

Quantitative SERS analysis of a certain analyte in a real sample in a fast and accurate way is the goal of developing SERS to be a routine analytical tool. In this section, we experimentally draw a three-step roadmap (Fig. 5): (1) realizing qualitative analysis with high sensitivity in the standard solution; (2) generating a reliable linear standard curve from the standard solution; (3) dealing with real samples with the aid of sample pre-treatment and data analysis.

#### 3.5.1 Qualitative analysis with a high sensitivity

There are two typical ways to perform quantitative SERS analysis: label and label-free methods. For the label method, the signal is from a known label with strong and well-characterized spectral features. It does not require too much effort to do qualitative analysis. However, for the label-free method, it is important to perform a rigorous qualitative analysis before the establishment of a reliable protocol for the quantitative analysis, because the peak position and relative peak intensity may vary for the ordinary Raman and SERS spectra and under different measuring environments (dry or solution) and at different concentrations. Two important considerations should be regarded:

##### (a) Ensure the reliability of the qualitative analysis

It is important to verify that the SERS signal is indeed from the analyte by performing: (i) analyte titration. The SERS signal should increase with the analyte concentration and may eventually saturate. This also facilitates the choice of characteristic Raman peaks for the quantitative analysis. (ii) Varying the laser power and illumination time. If the SERS spectral feature is independent of these two factors, it means the observed signal is from the analyte but not interference from potential photo-induced chemical reactions.

##### (b) Push to the required sensitivity

In practical applications, it is important to maximize the sensitivity by choosing the optimal condition of the substrate, the laser wavelength, and the measurement environment. (i) Gold and silver nanostructures are the most typical SERS substrates, with the former being chemically more stable. Although generally a higher EM enhancement is expected for silver than for gold, the limit of detection (LOD), or the lowest detectable concentration for a specific analyte, may be lower on gold if the analyte interacts more strongly with gold than silver and if the excitation laser is not optimized. Usually, gold can be used for excitation wavelengths from red to the near infrared region, and silver can be used over the whole visible to near infrared region. (ii) The optimal wavelength depends on the size, shape, arrangement, and aggregation state of the nanostructures and the measurement environment. Therefore, an appropriate substrate must be chosen according to the specific properties and the expected lowest detectable concentration of analytes. (iii) Normally, colloids present good sensitivity while solid substrates have good uniformity. For colloids, a suitable aggregation state must be set by adjusting the aggregating agent, pH, surface chemistry, and the volume ratio among the analyte, colloids and aggregating agent.^110^ For solid substrates, different sample conditions (wet or dry) can be chosen according to specific requirements.

#### 3.5.2 Generating a reliable linear standard curve

Quantitative analysis of the analyte can then be carried out with the knowledge obtained from the qualitative analysis. Typically, LOQ (or lowest quantifiable concentration), the linear range, and the linearity (quantitative accuracy) are the three parameters necessary for evaluating the quantification level of a technique. In general, the higher the demand on the quantification level, the more rigorousness is needed in the experimental design.

##### (a) Semi-quantitative analysis

Under the optimized qualitative analysis condition, a semi-quantitative analysis can be easily carried out according to the following three steps: (i) select the most intense Raman peak from the analyte spectrum and use its peak intensity for the quantitative analysis, as long as the FWHM remains constant at different concentrations. Otherwise, either use the peak area or selecting another intense Raman peak with constant FWHM. (ii) Create a standard curve by plotting the intensity (or area) *versus* the logarithm of concentration. This becomes a common practice in practical SERS analysis. Although such a plot may seem linear over a wide concentration range, its accuracy of quantification is relatively low. (iii) Determine the linear range of the intensity *versus* concentration with a proper adsorption model (such as Langmuir, as stated in the previous section), if a higher level of accuracy is demanded. However, usually the quantification level is still not high enough for a typical reproducibility of 20% for commercial substrates, as mentioned in Section 3.2.^5^

##### (b) Pushing to the limit of quantitative analysis

For precise quantification, a suitable internal standard strategy may be used for an effective calibration of SERS signals, as discussed in Section 3.4: (i) for traditional SERS substrates, it is advisable to use an internal standard that has similar chemical properties but different spectral features from those of the analyte. To avoid potential competitive adsorption, a suitable concentration of internal standards should be used. The concentration can be determined during the analyte titration. The linear range may be limited, due to the inevitable competitive adsorption at high concentrations; (ii) the CMS approach is preferred, in order to ensure the accuracy of the quantitative analysis. The CMS method can eliminate the interaction between the internal standard and the analyte.

#### 3.5.3 Dealing with real samples with the aid of sample pre-treatment and data analysis

With the standard curve in hand, quantitative analysis of the analyte in real samples can then be carried out with a proper sample pre-treatment and data processing.

##### (a) Sample pre-treatment for the practical application

The major factor or the first priority in limiting SERS for use in practical applications lies in how to realize the selective sensitivity down to the single molecule level towards trace target in complex media, like food, serum, or tissues. The concentrations of components and impurities from the matrix are usually several orders of magnitude higher than that of the analyte. These species can potentially cause non-specific adsorption onto the nanostructure and prevent the adsorption of the analytes, which affects the accuracy of quantitative analysis. Therefore, sample pre-treatment is an indispensable step for practical SERS trace analysis to diminish the detrimental interference of the non-specific adsorption of the complex matrix.^8^ In addition, it helps to enrich the analyte molecules and improve the signal-to-noise ratio. In comparison with traditional analytical methods for trace analysis in a complex matrix, such as GC-MS, the virtue of SERS is its capability for fast, on-site (or *in situ*) tests. In case of the emergency application or fast inspection, it is necessary to develop a fast and efficient pre-treatment method applicable to either a group of analytes or one analyte under a specific matrix. Under this condition, the high accuracy of the quantitative analysis of SERS has to be sacrificed to provide a rough estimation of the presence of a certain analyte.

##### (b) Data processing

The experimentally obtained SERS spectra may be noisy with a large background and the spectral difference may be trivial. Data processing of the raw spectra may include baseline correction, denoising, and identification. Outliers, such as cosmic rays, are easy to identify and thus removed from the data set *via* median filter. The background fluorescence contribution can be greatly reduced *via* polynomial fitting or adaptive iteratively reweighted penalized least squares (airPLS). Spectra with poor SNR can be improved with noise-reduction approaches such as the Savitzky–Golay (SG) algorithm or wavelet transform.^111^ Principle component analysis (PCA), cluster analysis, and multivariate curve resolution (MCR) can be used to identify the maximal chemical information from the spectra even without *a priori* knowledge of the chemical properties of the sample.^112^

## 4. Future developments

In the previous sections, we have discussed current and potential strategies for overcoming well-known limitations of SERS to improve the SERS performance for routine analysis. Such strategies are the result of a continued effort from the SERS community aimed at improving the reliability of this powerful technique. Thanks to this, the field has grown enormously since its discovery forty-years ago and caught the attention from diverse areas such as electrochemistry, materials, energy, life science, *etc.* Nevertheless, current-scientific research demands techniques to be able to analyze even more complex systems. To satisfy this requirement, SERS must be further improved in the following respects.

### 4.1 SERS substrates

High-performance SERS substrates are still the key factor limiting the wide application of SERS. This is reflected by the large number of reports on this aspect. However, different from 20 years ago, the current focus is on the development of SERS substrates with low signal fluctuation, low fluorescence or photoluminescence backgrounds, high uniformity and stability, high affinity to the analyte, and less photo-induced reaction or desorption. It is quite routine for a SERS substrate of silver or gold to have a SERS enhancement factor that is greater than 6 orders of magnitude. Therefore, it is now no longer the period to demonstrate only the high SERS enhancement while not considering the abovementioned issues affecting the practical application of SERS. Although there are reports about Raman enhancement from graphene,^113–115^ diverse 2D materials,^116^ and defective metal oxides, the EFs are generally not as high as those obtained with silver and gold substrates. It may be good to understand the SERS mechanism on these materials, but they may not be optimal substrates for practical analyses. However, there are interesting efforts on employing ultrathin layers of two dimensional (2D) materials, like graphene and transition metal dichalcogenides (TMD), as complementary platforms or shells on the conventional metallic SERS substrates. These materials can significantly enhance their affinity to the analyte molecules, which increases the surface molecular number and improves the detection sensitivity and selectivity. On the other hand, their atomic-scale thickness will not lead to much decay of the plasmonic field and compromise their enhancement. Therefore, these materials are ideal for being integrated with traditional SERS substrates.

For the traditional SERS substrates, there are increasing efforts towards the development of non-typical substrate configurations like nanoholes, gratings, or other nanoapertures, which may be advantageous for SERS dynamic sensing applications at the single molecule level, especially in the field of life science.^117,118^ We would also like to bring the readers' attention to the current efforts towards finding new materials with SERS enhancement approaching that of gold and silver. For example, Mo-doped Ta_2_O_5_, WO_3_ with oxygen vacancies, nanostructured TiN,^119,120^ and metal telluride have been observed to show SERS in the visible region,^39,44,119,121^ while having very good chemical stability. However, it remains challenging to understand the enhancement mechanism, and it requires rigorous evaluation of the SERS enhancement.

Nevertheless, with the good sensitivity ensured by SERS, we may further improve the spectral, temporal, and spatial resolution of the technique, so that we can obtain rich information from the systems in which we are interested.

### 4.2 Spectral resolution

An ideal Raman spectrometer can resolve individual peaks while retaining high sensitivity. The use of notch filters, especially volume Bragg gratings, single gratings, and charge-coupled devices (CCDs) has significantly improved the sensitivity and helped popularise Raman microscopy. Nevertheless, such a design still has limited spectral range and sensitivity in terms of photon efficiency and spectral resolution, which may be overcome by the implementation of dispersive echelle gratings, narrowband filters, and broadband filters. For instance, echelle gratings were used in Raman spectrometers to achieve high resolution in a more compact size and cover a much wider spectral range than conventional grating spectrometers. For the construction of miniaturized spectrometers without gratings, colloidal quantum dots (CQDs) were arranged into an array and placed in front of an arrayed detector and used as a broadband absorptive filter.^122^ The spectral resolution (2–3 nm) and spectral range can be simultaneously improved by increasing the number of CQDs in the array. However, high spectral resolution is still challenging to achieve in a miniaturized device. More recently, an ultracompact spectrometer was built, wherein a single semiconductor nanowire split and detected the light.^123^ The semiconductor nanowires were fabricated with different composition along their lengths, so that different segments had different absorptions. The generated photocurrent was cross-referenced with a pre-calibrated response, and the final spectrum was reconstructed computationally. Remarkably, this device depicted a spectral resolution similar to that of conventional spectrometers. If a two-dimensional array of nanowires can be fabricated, the hyperspectral information can be obtained over the whole surface, to simultaneously achieve spectral, temporal, and spatial resolution of a sample.

### 4.3 Temporal resolution

In conventional microscope-based techniques, the temporal resolution is limited by the frame rate of the detector array and by the mechanical speed of the optical setup. Nowadays, electron multiplied charged coupled device (EMCCD) technology reaches readout times down to a few milliseconds per spectrum without compromising data reliability. Additionally, line-scanning optical setups allow the possibility to perform Raman imaging in a few minutes.^124^ Certainly, SERS temporal resolution will be constantly furthered by improvements in instrumentation. However, there will always be room for the development of alternative strategies for pushing SERS towards the analysis of short-time-scale processes. As a starting point, it is possible to take advantage of the ease with which SERS can be coupled with other techniques. Some successful examples are the combination of confocal Raman and dark field microscopies for intracellular analysis on living cells,^125^ and the technique of transient electrochemical surface-enhanced Raman spectroscopy (TEC-SERS).^126^ The latter enables the analysis of the structural evolution of molecules participating in electrochemical processes with a time resolution on the order of milliseconds. Wide-field microscopes can carry out this task by expanding the laser-beam large enough to illuminate the whole sample at once. In Raman imaging, wide-field microscopy was demonstrated to largely reduce the total imaging time in comparison with the raster scan of typical optical setups.^127,128^

Alternatively, techniques of data processing based on machine learning may open new opportunities to improve the temporal resolution. A high-deep convolutional neural network (DCNN) assisted fast Raman imaging method was introduced to investigate living cells.^129^ By widening the slit and laser beam, the sample was scanned with a larger step than the one used in the typical fast-line scan Raman imaging. To improve the situation of reduced image quality due to the shorter imaging time, the spectral-data sets were processed *via* a DCNN regression approach to transform the low-resolution images into the high-resolution ones.

However, the characterization of transient species such as reaction intermediates is still challenging, since these are usually present at low concentrations and have weak signals and lifetimes ranging from picosecond to femtosecond timescales. These time scales are unreachable in the current–time resolution of SERS (milliseconds regime). Thus, SERS may be combined with pump-probe methods to improve its temporal resolution and expand its range of applications.^130–134^

### 4.4 Spatial resolution

SERS is an optical method, and its spatial resolution is roughly at the same level as that of a microscope: half wavelength limited by optical diffraction. By adapting the experimental protocols of super resolution fluorescence microscopy, the characteristic stochastic intensity fluctuations of SM-SERS have been exploited to construct high resolution images of SMs residing within hot spots.^135–137^ In this case, the centroid position was obtained by the point spread function to reveal the location of SMs in the hot spot and over the laser spot.^135^ This method makes use of the blinking of SM-SERS to avoid overlap between point spread functions from different hot spots. This can achieve a spatial resolution of a few nanometers. A SERS image of collagen fiber with 10 nm resolution was demonstrated by employing a plasmonic nanohole array and a laser optical diffuser to simultaneously excite different areas of the sample and thus obtain images without blank spots.^136^ Recently, high-speed super resolution SERS imaging was developed by taking advantage of signal fluctuations due to surface reconstruction of silver nanoparticles, super-resolution fitting, and an Airyscan detector.^138^ A point spread function from a single intensity fluctuation (SIF) occurring at a single nanoparticle fully-coated with an analyte is imaged by the Airyscan detector on the submillisecond time scale. Several SIFs with different spatial distributions are accumulated over a period of time and then fitted using a Gaussian function to reconstruct the image of the particle. The spatial resolution of this SERS imaging method can reach 7 nm, and the acquisition rate can reach 800 000 frames per second.

Furthermore, in Raman imaging, super resolution methods like structured line illumination Raman microscopy (SLI)^139^ and wide-field structured illumination^140^ were successfully developed. SLI was proven to improve the spatial resolution by a factor of 1.4 over that of confocal Raman microscopy. However, acquiring high quality images requires of long imaging times. This problem may be solved by using wide-field structured illumination rather than SLI.

On the other hand, the problem of limited-spatial resolution can be ultimately addressed by tip-enhanced Raman spectroscopy (TERS). This technique has been demonstrated to have a spatial resolution of 0.15 nm at low temperature and in the ultrahigh vacuum with simultaneous molecular fingerprint information.^141^ It can also be used for nanoscale characterization of different surfaces, solid–liquid interfaces,^142,143^ and even the electrochemical systems.^144–146^ However, the study of living cells or intracellular environment remains challenging, since the large size of these systems precludes work in the high enhancing configuration in which the SPR of the metallic substrate and the LSPR of the tip are coupled.

### 4.5 Study on ultrahigh vacuum and ultralow temperatures

Since conventional SERS studies are conducted under ambient temperature and pressure conditions, the state of investigated molecules is constantly changing on the nanostructure surface (*i.e.*, thermal movement, migration, configuration changes, oxidation or even photochemical reaction).^147^ By conducting SERS measurements in the ultra-high vacuum and at ultra-low temperature, it may be possible to comprehensively elucidate reaction mechanisms and enhancement mechanisms.

### 4.6 Combination with other related technologies

SERS is a near-field technique providing fingerprint structural information of large and small molecules in a nondestructive and label-free manner. These strengths of SERS can be hyphenated with other techniques to obtain more in-depth molecular information about the system to be studied. Therefore, it will be beneficial to integrate SERS with structure-related techniques like electron microscopy (EM), nuclear magnetic resonance (NMR), X-ray photoelectron spectroscopy (XPS), and X-ray diffraction (XRD) to synergistically gain correlated data so far precluded for technical reasons, including high-resolution images, three-dimensional structures of large components, atomic magnetic properties, molecular electronic state, atomic structure, *etc.* A major challenge of interfacing these techniques arises from the fact that these techniques work with different operation principles or require different sample conditions and preparation. However, if accomplished, insights so far hidden from conventional SERS will be of paramount importance for the field of chemistry in general.

## Conflicts of interest

The authors declare no competing financial interest.
